# PKCζ-dependent upregulation of p27kip1 contributes to oxidative stress induced retinal pigment epithelial cell multinucleation

**DOI:** 10.18632/aging.101299

**Published:** 2017-10-09

**Authors:** Dinusha Rajapakse, Mei Chen, Tim M. Curtis, Heping Xu

**Affiliations:** ^1^ Centre for Experimental Medicine, School of Medicine, Dentistry and Biomedical Sciences, Queen's University Belfast, Belfast, BT97 BL, UK

**Keywords:** aging, multinucleation, retinal pigment epithelium, protein kinase C, p27kip1

## Abstract

Retinal pigment epithelial (RPE) cells increase in size and multinucleate during aging. We have shown using human and mouse cell lines that oxidised photoreceptor outer segments (oxPOS)-induced cytokinesis failure is related to RPE cell multinucleation, although the underlying mechanism remains unknown. This study investigated the role of the PKC pathway in oxPOS-induced RPE multinucleation using ARPE19 cells. oxPOS treatment promoted PKC activity and upregulated the mRNA expression of PKC α, δ, ζ, ι and μ. Inhibition of PKCα with Gö6976 resulted in a 33% reduction of multinucleate ARPE19 cells, whereas inhibition of PKCζ with Gö6983 led to a 50% reduction in multinucleate ARPE19 cells. Furthermore, oxPOS treatment induced a PKCζ-dependent upregulation of the Cdk inhibitor p27kip1, its inhibition using A2CE reduced oxPOS-induced ARPE19 multinucleation. Our results suggest that oxPOS-induced ARPE19 cytokinesis failure is, at least in part, due to the upregulation of p27kip1 through activating the PKC, particularly PKCζ pathway. Targeting the PKCζ-p27kip1 signalling axis may be a novel approach to restore RPE repair capacity during aging.

## INTRODUCTION

Multinucleate cells, i.e., multiple nuclei share one common cytoplasm, are frequently observed in various patho-physiological conditions, including development, aging, inflammation and malignant tumor. Multi-nucleate cells can be induced by the fusion of multiple cells or formed by nuclear division that is not followed by cytokinesis [1]. During inflammation, such as infection from tuberculosis, herpes, HIV, or foreign bodies, macrophages can be multinucleated [2]. Inflammation-induced microglia multinucleation is known to be due to cytokinesis failure [3]. Age-related multinucleate cells have been observed in various tissues and cells, such as vascular endothelial cells [4] and retinal pigment epithelial (RPE) cells [5].

The appearance of binuclear and multinuclear RPE cells in mice and humans are mostly reported in ageing and disease conditions such as age-related macular degeneration (AMD). Ts'o and Friedman's landmark studies in the late 1960′s noted a variable increase in RPE cell size as well as multinucleation with age in humans [5]. Al-Hussaini et al further reported multinucleate RPE cells in proximity to drusen and they are greater in number in AMD compared to age matched healthy controls [6]. In rodents, binucleation is a late developmental event with 2% of cell binucleated at P1 and 26% by P30 in mouse [7]. We reported an age-dependent increase in the number and size of multinucleate RPE cells in mice [8]. However, the underlying mechanism related to age-induced multi-nucleation of RPE cells remains poorly defined. There is much debate on the mitotic ability of RPE cells. Del Priore [9] showed that there is little evidence for overall cell loss in the human RPE with age [9] and Al Hussaini [10] observed few dividing RPE in rats with BrdU. Whereas other studies show RPE cells declines in numbers with increasing age [8,11].

Phagocytosis of photoreceptor outer segments (POS) is essential for visual function. In our previous study, we have shown that multinucleation of RPE is due to cytokinesis failure mediated by POS, particularly the oxidized POS (oxPOS), through generation of reactive oxygen species (ROS) [8]. ROS are known to play an important role in several signalling pathways and cellular functions [12]. They have been implicated, for example, in the activation of different Protein Kinase C (PKC) isoforms [13]. Our system represents a model of oxidative insult-induced cell multinucleation under aging conditions.

PKC plays a crucial role in key cellular processes, including proliferation, differentiation, and mitosis [14]. There are three sub-families of PKC isoforms. The classic PKCs (cPKC: PKCα, PKCβI, PKCβII, and PKCγ) require calcium, phosphatidylserine, and diacylglycerol (DAG) for activation. The novel PKCs (nPKC: PKCδ, PKCε, PKCη, PKCθ and PKCμ) do not require calcium for activation. The activation of atypical PKCs (aPKC: PKCζ, PKCι) depends on phosphatidyl-serine, but not DAG or calcium [15]. The functional differences of different PKC isoforms are primarily due to their subcellular localisation, activation or inhibition by different stimuli. PKC isoform activation in RPE cells is well documented and has been shown to impact RPE cell migration, melanin synthesis and phagocytosis [16, 17]. Activation of PKCα isoform is known to be related to RPE proliferation and inhibition of PKCα has been considered as a potential therapeutic option for proliferative vitreoretinopathies (PVR) [18].

In this study, we investigated the role of the PKC pathway in oxPOS-induced RPE multinucleation. Our results demonstrate that oxPOS increases PKC mRNA expression and PKC activity in human RPE cells. Importantly, we show that blockade of PKC activity, particularly the PKCζ isoform of the atypical PKC subfamily suppressed oxPOS-induced RPE multi-nucleation.

## RESULTS

### oxPOS activates PKC and PKC activation is involved in RPE multinucleation

**Figure 1:**
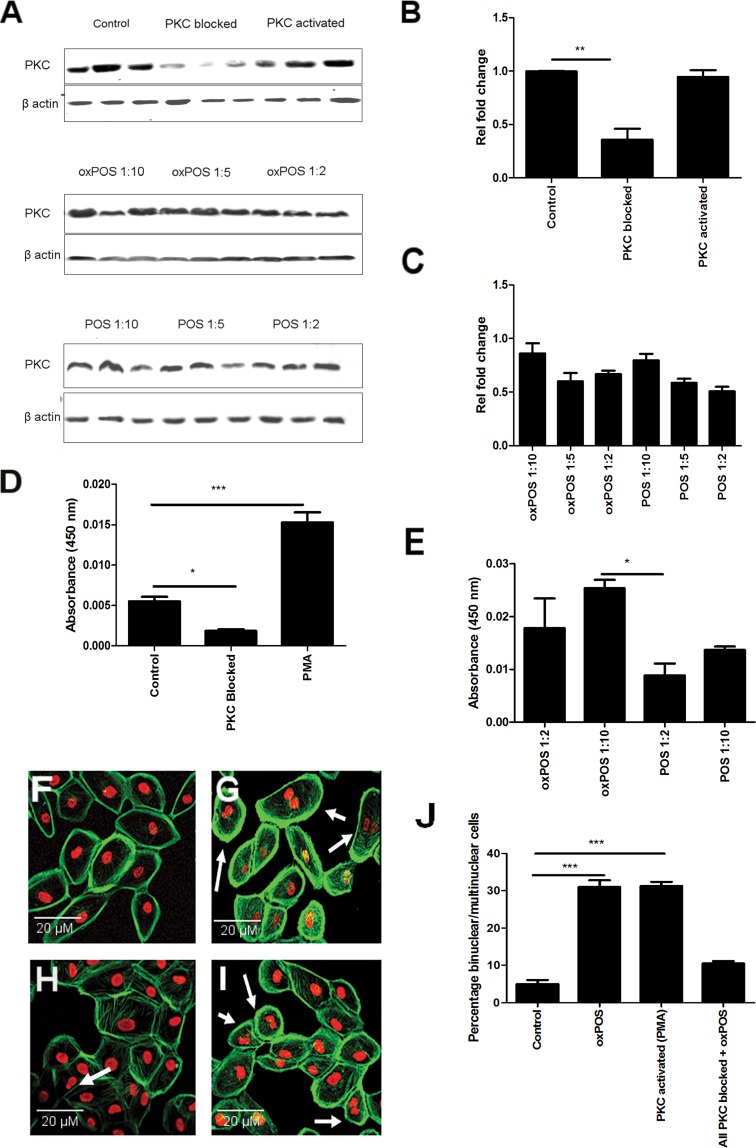
Role of PKC in RPE cell multinucleation and oxPOS induced PKC activation in RPE cells (**A**) Representative Western blots showing total PKC protein expression in RPE cells following PMA, total PKC blockade, no treatment and with different concentrations of POS or oxPOS treatments (RPE: POS/oxPOS 1:2, 1:5, 1:10) for 48h. (**B**-**C**) quantification of total PKC expression in different treatment groups. The data are represented as relative fold change to the control. (**D**) Active PKC levels in RPE cells following PMA, total PKC blockade treatments, or untreated control for 48h. (**E**) Active PKC protein levels in RPE cells following POS or oxPOS treatment at 1:10 and 1:2 (RPE: POS/oxPOS) for 48h. Cells from each treatment group was homogenised and protein levels were evaluated by active PKC ELISA kit. *, P < 0.05; ***, P < 0.001 compared to the control untreated group. One-way ANOVA followed by Dunnett's multiple comparison test. N = 3. (**F**-**I**): histochemical staining of propidium iodide (PI) and Phalloidin in control untreated ARPE19 (**F**), PMA treated ARPE19 (**G**), PKC blocked ARPE19 (**H**) and oxPOS (**I**) treated ARPE19 cells. Arrows indicate multinucleate RPE cells. (**J**) histogram showing the percentage of binucleated and multinucleate RPE cells, ***, P < 0.001 compared to control untreated. One-way ANOVA followed by Dunnett's multiple comparison test. 50 cells were counted from three wells for each group.

Western blot showed that PKC proteins are constitutive-ly expressed in ARPE19 cells (Fig. 1A). PMA, a PKC activator, and POS or oxPOS did not affect the expression of total PKC protein (Fig. 1A-1C), although the expression was suppressed by a cocktail of PKC isoform inhibitors (Fig. 1A, 1B). Interestingly, the PKC kinase activity was significantly enhanced by PMA (100 nM, Fig. 1D) and oxPOS in a concentration-dependent manner (Fig. 1E). Both PMA and oxPOS increased the percentage of multinucleate ARPE19 cells from 7% (under non-stimulatory conditions) to approximately 33%, an effect that was abolished by the PKC inhibitor cocktail (Fig. 1F-J). Comparable results were observed when the experiment was performed in human RPE cells derived from primary culture (Fig. S1). Our results suggest that PKC activation can induce RPE multinucleation.

### PKC isoform expression in POS and oxPOS treated ARPE19 cells

**Figure 2:**
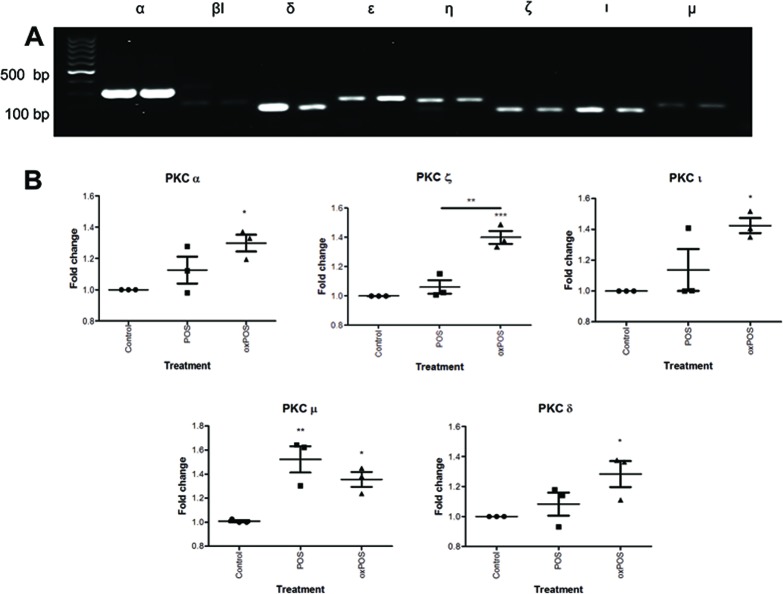

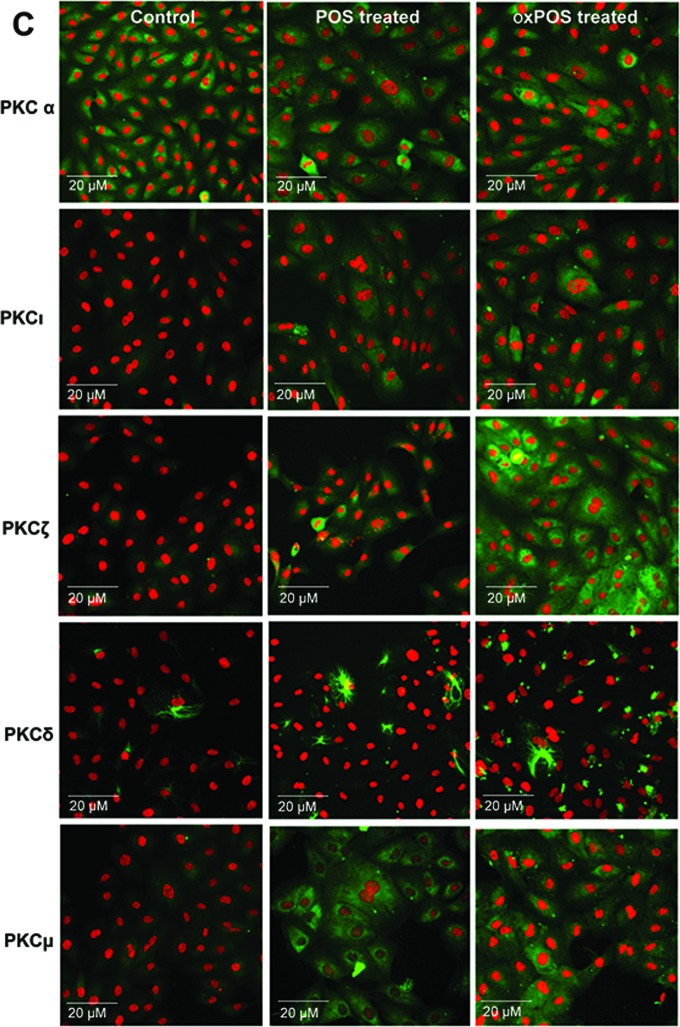
Expression of PKC isoforms in ARPE19 cells (**A**) Conventional PCR gel image showing expression of eight PKC isoforms-PKCα, PKCβI, PKCδ, PKCε, PKCη, PKCζ, PKCι and PKCμ in ARPE19 cells. (**B**) Real-time RT-PCR analysis of PKC isoforms in ARPE19 cells following POS, oxPOS treatments and untreated control. *, P < 0.05; **, P < 0.01, ***, P < 0.001 compared to the control untreated group. One-way ANOVA followed by Dunnett's multiple comparison test. N = 3. (**C**) Immunofluorescent staining of PKCα, PKCδ, PKCζ, PKCι and PKCμ isoforms in RPE cells following POS or oxPOS treatments. The isoforms were in green, nuclei were labelled by PI in red.

To understand which isoforms of PKC may be involved in oxPOS mediated RPE multinucleation, we examined the expression profile of different PKCs in ARPE19 cells. Of the 11 PKC isoforms from the three PKC sub-families, mRNAs for PKCα β1, δ, ε, ζ, η, ι, and μ isoforms were detected in ARPE19 cells (Fig. 2A). The other isoforms including PKC βII, γ and θ were not detected (Fig. S2), though they were detected in positive controls (human serum cDNA, data not shown). The treatment of ARPE19 cells with oxPOS significantly upregulated the expression of PKC α, δ, ζ, ι and μ compared to untreated controls (Fig. 2B), whereas POS treatment only enhanced the expression of PKC μ (Fig. 2B). Immunocytochemistry further confirmed the expression of PKC α, δ, ζ, ι and μ isoforms in ARPE19 cells under control, POS and oxPOS treated conditions (Fig. 2C).

### The role of PKCα in oxPOS-mediated RPE multinucleation

**Figure 3:**
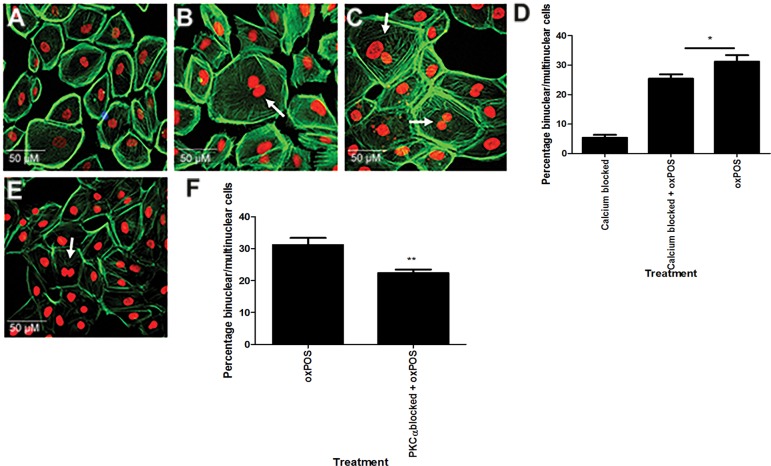
Involvement of Ca2+ signalling in RPE cell multinucleation Intracellular calcium was blocked in ARPE-19 cells using BAPTA-AM. (**A**) calcium blocked cells. (**B**) calcium blocked + oxPOS treated cells. (**C**) oxPOS treated cells. Arrows indicate multinucleate RPE cells. (**D**) histogram showing the percentage of binucleate and multinucleate RPE cells following calcium chelation *, P < 0.05, **, P < 0.01, ***, P < 0.001 compared to oxPOS treated group. One-way ANOVA followed by Dunnett's multiple comparison test. 50 cells were counted from three wells for each group. (E) PKCα inhibitor-treated RPE cells. (F) histogram showing the percentage of binucleate and multinucleate RPE cells after PKCα blockade **, P < 0.01 compared to oxPOS treated group. 50 cells were counted from three wells for each group.

PKCα is constitutively expressed by RPE cells at high levels (Fig. 2A, S2). PKCα belongs to the classic PKC family and its activation is calcium dependent, whereas the novel and the atypical family isoforms are calcium independent [19]. Intracellular calcium labelling using Fluo-4 revealed intense fluorescence in control untreated and oxPOS-treated ARPE19 cells (Fig. S3A, S3B). The treatment with BAPTA-AM (a Ca2+ selective chelator) significantly reduced Fluo-4 staining in oxPOS-treated cells and almost completed abolished the staining in untreated control cells (Fig. S3), suggesting that BAPTA-AM satisfactory blocked intracellular calcium in ARPE19 cells. Chelation of intracellular Ca2+ using BAPTA-AM 48h before oxPOS treatment led to a 33% reduction in the number of multinucleate cells (Fig. 3A-D). Similar effects were observed following treatment of the cells with a PKCα inhibitor (Fig. 3E-F). The results suggest that PKCα is partially responsible for oxPOS-induced ARPE19 multinucleation.

### The role of PKC δ, ζ, ι and μ in oxPOS-induced RPE multinucleation

**Figure 4:**
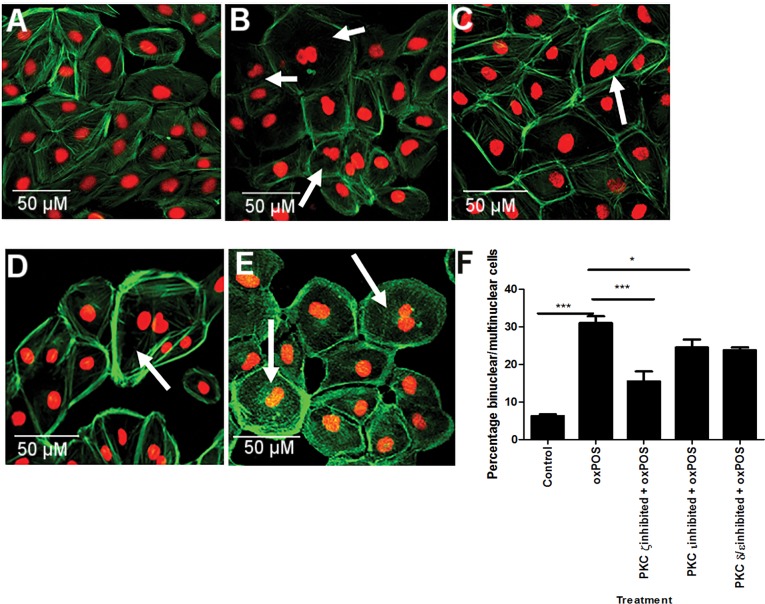
The effects of PKC isoform inhibition in RPE cell multinucleation (**A**) control RPE cells. (**B**) oxPOS treated RPE cells. (**C**) PKCζ inhibited + oxPOS treated RPE cells. (**D**) PKCι inhibited + oxPOS treated RPE cells. (**E**) PKC δ/ε inhibited + oxPOS treated RPE cells. Arrows indicate multinucleate RPE cells. (**F**) histogram showing the percentage of binucleate/multinucleate RPE cells following different treatments *, P < 0.05, ***, P < 0.001 compared to oxPOS treated group. One-way ANOVA followed by Tukey's multiple comparison test. 50 cells were counted from three wells for each group.

To further explore the role of different PKC isoforms on oxPOS-induced RPE cell multinucleation, the activities of other isoforms including PKC δ/ε, ζ, and ι that showed significant upregulation upon oxPOS treatment were blocked using specific inhibitors. PKCι inhibition resulted in 18% reduction in multinucleate cells (P<0.05, Fig. 4A, 4F). Interestingly, PKCζ blockade resulted in 50% reduction in multinucleate cells (Fig. 4B, 4F). Inhibition of PKC δ/ε isoforms did not significantly affect oxPOS-induced ARPE19 cell multinucleation (Fig. 4C, 4F). Our results suggest that PKCζ, a member of the atypical PKC subfamily, plays an important role in oxPOS-induced RPE multi-nucleation.

### PKC ζ-dependent expression of p27kip1 in ARPE19 cells

**Figure 5:**
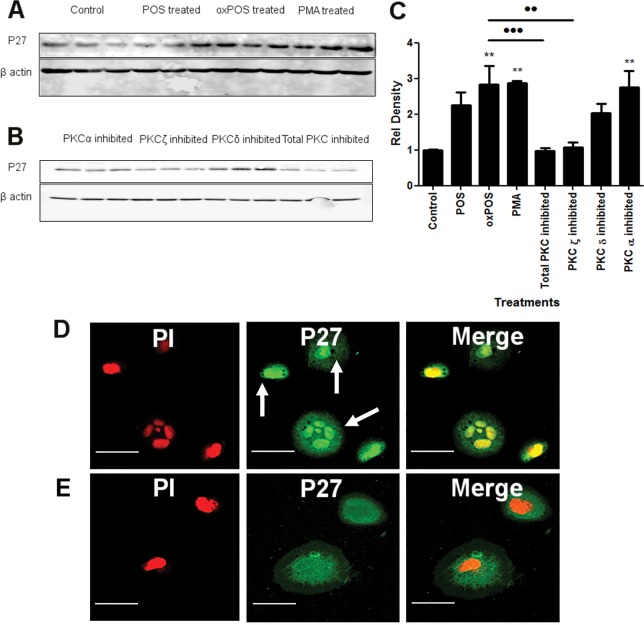
The effect of PKC inhibition on P27kip1 expression in RPE cells (**A**-**B**) representative Western blots from control, POS, oxPOS, PKCα, ζ, δ and total PKC inhibitor treated RPE cells showing p27kip1 at 28 KDa and β-actin at 40 KDa. (**C**) quantification of p27kip1 expression by RPE cells in the different groups. Data were expressed as relative fold change to the control. *, P < 0.05; **, P < 0.01, ***, P < 0.001 compared to the control untreated group and ··, P < 0.01 compared to oxPOS treated group. One-way ANOVA followed by Tukey's multiple comparison test. N = 3. (**D**) ARPE-19 cells were stained for p27kip1 (green) and PI (red) after 48 h oxPOS treatment. Arrows shows strong p27kip1 staining in cell nucleus. (**E**) control ARPE-19 cells stained for p27kip1 (green) and PI (red) showing weak, diffused p27kip1 (green) in the cytoplasm and nucleus.

PKC activity has been positively associated with phosphorylation of cell cycle regulatory molecules including Cdk inhibitory proteins p21 WAF1 and p27kip1. To further understand the underlying mechanism related to PKC mediated RPE multinucleation, we examined p21 WAF1 and p27kip1 expression in ARPE19 cells. A significant increase in p27kip1 protein expression was observed in oxPOS-treated group as well as the PMA-treated group (Fig. 5A), although the expression of p21 WAF1 was not affected by POS or oxPOS treatment (Fig. S4). The upregulation of p27kip1 in oxPOS treated ARPE19 cells was significantly reduced by PKCζ inhibitor (Fig. 5B, 5C), but not by the blockers specific to PKC α or δ (Fig. 5B, 5C). These results suggest that the upregulation of p27kip1 following oxPOS treatment depends on PKCζ activation.

Immunocytochemistry revealed strong p27kip1 expression in both the nucleus and cytoplasm in oxPOS treated ARPE19 cells (Fig. 5D), whereas only weak p27kip1 expression was detected in untreated ARPE19 cells (Fig. 5E).

### The role of p27kip1 in RPE cell multinucleation

**Figure 6:**
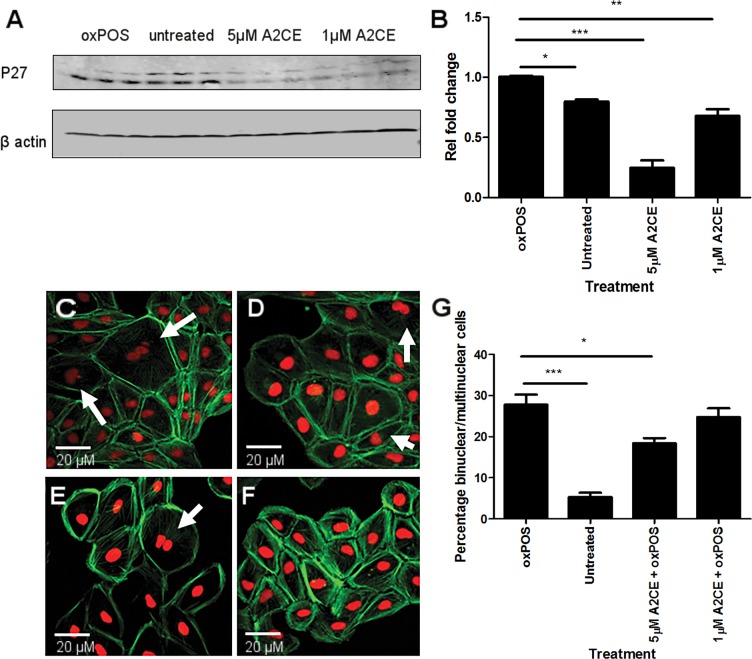
Inhibition of p27kip1 using Alsterpaullone, 2-Cyanoethyl (A2CE) and its effect on RPE multinucleation (**A**) representative western blot from oxPOS, control, 5μM A2CE and 1μM A2CE treated RPE cells showing p27kip1 at 28 KDa and β-actin at 40 KDa. (**B**) quantification of p27kip1 protein expression by RPE cells in the different treatment groups. Signals for POS treated group were set to one and the data are represented as relative fold change to the POS group. * P <0.05, *** P <0.001 compared to the POS group. One-way ANOVA followed by Tukey's multiple comparison test. N= 3. C-F: confocal images of multinucleate RPE cells in different treatment groups (**C**); POS-treated, (**D**); 1μM A2CE, (**E**); untreated RPE cells, (**F**); 5μM A2CE). (**G**) histogram showing the percentage of binucleate/multinucleate RPE cells following different treatments, * P <0.05, *** P <0.001 compared to oxPOS treated group. Data comprised of percentage of multinucleate cells per well from 5 different well for each group.

To further confirm the role of PKCζ - p27kip1pathway in oxPOS-induced RPE multinucleation, a p27kip1 transcriptional inhibitor, Alsterpaullone, 2-Cyanoethyl (A2CE) was used. A2CE inhibits p27Kip1 transcription by preventing FoxO3a from binding to the p27kip1 promoter [20]. A2CE at 1μM and 5μM did not show any toxicity to ARPE19 cells (data not shown). However, it dose-dependently suppressed p27kip1 expression (Fig. 6A, 6B), and reduced oxPOS-induced ARPE19 multinucleation (Fig. 6C, 6D). Our results suggest that p27kip1 is critically involved in oxPOS-induced RPE multinucleation.

## DISCUSSION

Previously, we have shown that POS and oxPOS increased ROS production and DNA oxidation in RPE cells, and we postulate that oxPOS-induced DNA oxidation may contribute to RPE multinucleation [8]. In this study, we further show that oxPOS activates the PKC pathway, particularly PKCζ, PKCα, and PKCι, leading to the upregulation of p27kip1 and cell cycle disruption. Our data suggest that the PKCζ - p27kip1 pathway may play a key role in oxidative stress mediated RPE cell multinucleation.

The PKC pathway is known to be involved in different types of cell multinucleation. Macrophages fuse to become multinucleate giant cells during chronic inflammation [21]. Cytokines such as IL-4, IL-13, IL-3, IL-17A, and interferon (IFN)-γ have been shown to induce macrophage multinucleation [22, 23]. The PKC pathways in particular PKCα, PKCβ, and PKCδ are known to be involved in IL-4-induced macrophage multinucleation [24, 25]. PKCs are also known to be involved microglia multinucleation under inflammatory conditions [3]. Various PKCs are known to be activated in microglia by Lipopolysaccharide (LPS), Amyloid beta (Aβ) and INF-γ [3]. Hornik and colleagues demonstrated that the PKC activator PMA increased microglial multinucleation whereas the PKC inhibitor Gö6976, inhibits PKCs α, β and ι, prevented LPS and Aβ-induced multinucleation. PMA has been shown to induce multinucleation of mesenchymal stem cells through inhibition of cytokinesis [26].

The role of ROS in the activation of PKCs is well established [27, 28]. In the current study, the level of active PKC was increased following oxPOS but not POS treatment. Interestingly, the treatment did not affect the total PKC protein levels although the mRNA expressions of some isoforms such as PKCα, PKCδ, PKCζ, PKCι and PKCμ were upregulated. When using specific inhibitors, the greatest suppression of multinucleate ARPE19 was observed when PKCζ was inhibited, followed by PKCα and PKCι inhibition, suggesting that these isoforms are involved in oxPOS-induced RPE multinucleation. The PKC proteins consist of two pairs of zinc finger structures within the regulatory domain and these are the sites of diacylglycerol (DAG) and phorbol ester binding. Each zinc finger is formed by a structure that consists of six cysteine residues and two zinc atoms [29], and the structure can be destroyed by oxidants, resulting in the catalytic activation of PKC in the absence of Ca2+ or phospholipids [30]. Oxidants can also modify the C-terminus of the catalytic domain leading to inactivation of PKC. This effect of oxidants and the resulting signalling mechanism vary in different isoforms and cell types [31]. We found that when Ca2+ is chelated the oxPOS-induced RPE multinucleation was only reduced by 30%, suggesting that oxPOS-mediated ROS may also act on the regulatory domain of the PKC and result in continuous PKC activity in the absence of Ca2+.

PKCs play important roles in cell cycle regulation. PKC regulates cell cycle both in G1/S and in G2/M stages through the modulation of CDK activity [32]. However, the precise role of different isoforms of PKC in the control of cell cycle and the complex intracellular networks involved are poorly explored. A previous study has shown that PKCα plays a predominantly inhibitory role in cell cycle progression in intestinal epithelial cells [33]. The authors demonstrated that treatment of IEC-18 cells with a panel of PKC agonists resulted in G1 arrest and delayed transition through the S and G2/M phases through upregulation of p21 and p27, which then lead to hypophosphorylation of retinoblastoma protein [33]. Here, we show that PKCζ activation plays an important role in oxPOS-induced cytokinesis failure and multinucleation in RPE cells through upregulating p27kip1 expression. Our study is the first to demonstrate that the activation of PKCζ exerts effects on the p27kip1 to induce cell multinucleation.

The p27kip1 is known to be part of a cell-intrinsic regulator that arrests cell cycle and initiates differentiation [34]. Our findings mirror those reported in previous studies investigating PKC-induced upregulation of p27 in other cell types [35, 36], although in different down-stream pathways. A previous study has shown that PKCα mediated down-regulation of p27kip1 promoted RPE proliferation, whereas p27kip1 up-regulation resulted in suppressed RPE proliferation [18]. Other studies have shown that p27kip1 knockout led to increased capacity of RPE to proliferate [37, 38]. Kokkinopoulos and colleagues found that the p27kip1 was significantly elevated in mouse RPE cells from the central regions of the eye-cup in comparison to cells from the periphery [39]. This geographic-specific upregulation of p27kip1 is in line with the distribution of bi-/multi-nucleate RPE cells in adult/aged mice [8] and aged human eyes [40]. Yoshida and colleagues [38] demonstrated up-regulation of p27kip1 expression when RPE was detached from the retina. Furthermore, Defoe and colleagues [37] found that the average size of individual RPE cells was significantly smaller in p27kip1 null mice, compared to wild-type litter mates. These data suggest that p27kip1 is an important regulator of cell proliferation at least in RPE cells. In the current study, it was demonstrated that p27kip1 was implicated in RPE cell multinucleation, a concept already evidenced in other cell types including myogenic cells and osteoclasts [41, 42].

RPE has been considered as a post-mitotic cell and RPE senescent and dysfunction underlines many age-related retinal degenerative diseases including AMD [43]. We argue that RPE nuclei are capable of duplication in response to stimuli in vivo. However, the full cycle of cell division cannot be completed due to oxPOS mediated cytokinesis failure [8]. This study uncovered a novel signalling pathway related to oxidative insult-mediated cell multinucleation highlighting the roles of PKCs, in particular PKCζ isoform and Cdk inhibitor p27kip1 (Fig. S5). PKC activation not only is associated with fusion (macrophage) and inflammation-induced (microglia) cell multinucleation, but also functions as regulator in oxidation-induced cell multinucleation.

Further understanding the molecular mechanism related to oxPOS-induced PKC-p27kip1 activation may uncover novel targets to normalize RPE cell cycle and promote in vivo cellular repair. This could represent a novel approach to prevent or treat RPE dysfunction during retinal degenerative diseases such as AMD. ROS-mediated cell cycle disruption and cytokinesis failure may also be responsible for the multinucleation of other cells under aging or inflammatory conditions. The role of the PKC-p27 kip1 pathway in various age-related cell multinucleation warrants further invest-tigation.

## MATERIALS AND METHODS

### In vitro RPE cell culture

Human ARPE19 cells were purchased from ATCC (CRL-2302, Middlesex, UK). Primary human RPE cells were cultured from a donor eye (free of known eye diseases) obtained from the Manchester Eye Bank using the protocol described previously [44]. Briefly, after the removal of vitreous and retina, the eye cup was washed with Hanks’ balanced salt solution (HBSS), and filled with 0.25% (v/v) trypsin (ThermoFisher, Hemel Hempstead, UK) for 2h at 37°C. Cells were then removed from the basement membrane by gentle aspiration. The cells were washed twice in Dulbecco's Modification of Eagle's Medium F12 (DMEM F12, GIBCO BRL, Paisley, UK). Both ARPE19 cell and primary human RPE cells were cultured in complete DMEM F12 medium containing 10% fetal calf serum (FCS) and 100 μg/mL Primocin (Invitrogen, San Diego, California, USA). Cells were maintained at 37°C with 5% CO2. The phenotype of RPE cells were confirmed by pan-cytokeratin staining. The use of donated human eye tissue was approved by local Research Ethics Committee. During all cell culture experiments, the RPE cell line used were maintained between passages 3 to 10 to ensure that RPE cell morphology had not changed or transdifferentiated. When inducing multi-nucleate RPE cells, cell confluency was maintained at approximately 70% to ensure sufficient number of proliferating cells. This also facilitates identifying individual multinucleate cells in culture and omitting false-positive of overlapping cells.

### Preparation of photoreceptor outer segments

POS were isolated from bovine eyes using method described previously [45]. Briefly, 10 bovine retinas were placed in 10 ml homogenising solution (20% w/v sucrose, 20 mM Tris acetate, pH 7.2, 2 mM MgCl2, 10 mM glucose and 5 mM taurine). The suspension was shaken gently for 1min and then filtered through a 100 mm cell strainer (BD, Oxford, UK) to remove tissue debris. The suspension was layered on 25∼60% w/v continuous sucrose gradients containing 20 mM Tris acetate pH 7.2, 10 mM glucose and 5 mM taurine and centrifuged at 25,000 rpm for 45min at 4°C. The pink band containing the POS was collected and washed with storage buffer (10 mM sodium phosphate, pH 7.2, 0.1 M NaCl and 2.5% sucrose). For storage, isolated POS aliquots were stored at −80°C at a concentration of 10^8^ POS/ml. The oxidized POS (oxPOS) were generated by exposing POS to 302-nm ultraviolet light (Ultraviolet Products, Cambridge, UK) in a laminar air-flow box for 12h [45].

### In vitro generation of multinucleate RPE cells

Multinucleate RPE cells were generated by incubating the cells with POS or oxPOS as previous described [8]. In brief, ARPE19 and human RPE cells were seeded at a density of 3000 cells per well in 12-well plates and incubated for 5h. The cells were then treated with POS or oxPOS for 48h (RPE: POS = 1:5).

### PKC inhibitor experiments

ARPE19 cells incubated with/without oxPOS were treated with PKC inhibitors (Gö 6983, inhibitor of PKCζ at IC50 = 60 nM; Gö 6976, inhibitor of PKCα at IC50 = 10 nM; Aurothiomalate (ATM), inhibitor of PKCε at IC50 = 132 nM; Bisindolylmaleimide I, inhibitor of PKCι at IC50 = 1μM as recommended by manufacturer) for 1h followed by further 48h incubation with POS or oxPOS (RPE: POS = 1:5). The inhibitors were purchased from either Sigma Aldrich, Cheshire, UK (Gö 6983, Gö 6976, Bisindolylmaleimide I) or Cambridge Bioscience, Cambridge, UK (ATM).

### Calcium chelator studies

ARPE19 cells were treated with the 10 μM intracellular Ca2+ chelator BAPTA-AM (ThermoFisher, Hemel Hempstead, UK) for 30 min at 37°C. The cells were washed and incubated with POS or oxPOS for 48h. Intracellular Ca2+ staining was performed by incubating cells with 5μM Flou-4 (ThermoFisher, Hemel Hempstead, UK) for 60min at 37°C. The cells were washed thoroughly before imaging with confocal microscope (Eclipse TE2000-U, Nikon, Surrey, UK) at 10X magnification, numerical aperture 0.25.

### Western blot

RPE cells were lysed in RIPA buffer with protease inhibitors (Sigma-Aldrich, UK). The protein concentrations were measured using a BCA protein assay kit (PIERCE, Cramlington, UK). 20μg of the total protein was loaded into 10% SDS-PAGE gel. Gels were run at 80V for 30 min followed by 150V for 60min. Proteins were transferred to the Immobilon-FL polyvinylidene difluoride (PVDF) membrane (Millipore, Watford, UK) at 350 mA for 50 min. The membranes were blocked with 5% bovine serum albumin (BSA) in tris-buffer saline with tween 20 (TBS/T) for 1h at room temperature, followed by incubation with primary antibodies at 1:1000 dilutions at 4°C overnight. After thorough washes, the membranes were incubated with secondary antibodies at 1:5000 dilutions for 2h in dark at room temperature. The following primary antibodies were used for western blot experiments: PKC (1:1000; Cell Signaling), p27kip1 (1:1000; Life Technologies), p21 (1:1000; Life Technologies), β-actin (1:1000; Santa Cruz). The secondary antibodies used were: Goat anti-rabbit IRDye 680 (1:5000), Goat anti-rabbit IRDye 800 (1:5000), Goat anti-mouse IRDye 680 (1:5000), Goat anti-mouse IRDye 800 (1:5000); (LI-COR; Lincoln).

The membrane was then washed in TBS/T 3 times before scanning using Odyssey infrared imaging system (Li-COR Biotechnology, Cambridge, UK). Quantitative western blotting was performed using imageJ software (version 1.45).

### PKC kinase activity assay

Active PKC levels in ARPE19 cells were measured using a commercially available kit (ab139437, Abcam, Cambridge, UK) in accordance with the manufacturer's instructions. Briefly, 30μg/30μL protein of each sample was added to an active PKC capturing antibody coated 96-well plate (Abcam) which was then incubated at 30°C for 90min. 40μL of the phosphospecific substrate antibody was added to each well. After a single wash, 40μL of diluted anti-rabbit IgG: HRP conjugate was added to each well and incubated at room temperature for 30 min. Following another single wash, 60μL TMB substrate was added to each well and left for 30min. The assay was terminated by addition of 20μL stop buffer and absorbance measured immediately at 450nm using a microplate reader (BMG Labtech, Ortenberg, Germany).

### Real-time polymerase chain reaction (real-time RT-PCR)

Total RNA was extracted from RPE cells using Tri Reagent (Sigma-Aldrich, Cheshire, UK). The quantity and quality of the RNA was determined by Nano-Drop ND-1000 spectrophotometer (NanoDrop Technologies, Wilmington, DE). cDNA synthesis was performed in a reaction of 0.5-2.5μg of total RNA with a random primer using the SuperScript™ II Reverse Transcriptase kit (Invitrogen, Paisley, UK). Real-time RT-PCR was performed in 384-well plates using the LightCycler 480 system (Roche Applied Science, Mannheim, Germany). The reaction mixture contained 6μL of LightCycler 480 SYBR Green Master (Roche Diagnostics GmbH, Mannheim, Germany), 0.5μM primers and diluted cDNA to make a total volume of 12 μL. The primers used were purchased from Integrated DNA Technologies UK, Ltd and are listed in (Table 1). Real-time RT-PCR quantifications were run in triplicates for each sample. PCR products were quantified by the LightCycler 480 software. Expression levels were normalised to 18S. Gene fold changes of treated group against untreated control group were calculated by dividing the gene expression levels of treated samples by control samples. PCR products were electrophoresed on 2% agarose gel in TAE buffer to confirm product size.

**Table 1:** PCR primers for PKC isoforms

Family	Subtype	Primer Deoxy ribonucleotide sequences	Predicted size (base pairs)
cPKC	PKCα	Forward 5′CGACTGTCTGTAGAAATCTGG3′	327
		Reverse 5′CACCATGGTGCACTCCACGTC3′	
	PKCβI	Forward 5′TTGTGATGGAGTATGTGAACGGG3′	404
		Reverse 5′CCTGGGTGTTTGGTCATTAGCC3′	
	PKCβII	Forward 5′GACCGGTTTTTCACCCGCCA3′	409
		Reverse 5′GGCATTTTCTCTCCCCATTGG3′	
	PKCγ	Forward 5′TTGATGGGGAAGATGAGGAGG3′	233
		Reverse 5′GAAATCAGCTTGGTCGATGCTG3′	
nPKC	PKCδ	Forward 5′CACCATCTTCCAGAAAGAACG3′	189
		Reverse 5′CTTGCCATAGGTCCCGTTGTTG3′	
	PKCε	Forward 5′AGCTTGAAGCCCACAGCCTG3′	249
		Reverse 5′CTTGTGGCCGTTGACCTGATG3′	
	PKCη	Forward 5′CCATGAAGATGCCAGAGGGATC3′	239
		Reverse 5′TCATCAATCGGAGTTAAGACGGG3′	
	PKCθ	Forward 5′CTATCAATAGCCGAGAAACCATG3′	250
		Reverse 5′CTCATCCAACGGAGACTCCC3′	
aPKC	PKCζ	Forward 5′CGATGGGGTGGATGGGATCAAAA3′	166
		Reverse 5′GCAGAAAGTGCTCGTTGTGTCAC3′	
	PKCι	Forward 5′TATAATCCTTCAAGTCATG3′	206
		Reverse 5′TTACACATGCCGTAGTCAGT3′	
PKD	PKCμ	Forward 5′CATTGGCGAGAAGTCTTTCCG3′	134
		Reverse 5′TCAGGCTCACATAGATGATGACCC3′	

### Immunofluorescence staining

ARPE19 and human RPE cells derived from primary culture were cultured on cover slips and treated with POS or oxPOS for 48h. Cells on cover slips were washed with cold PBS and fixed with 1% para-formaldehyde (PFA) for 10 min, followed by permeabilisation with 0.1% Triton-X for 5 min. The samples were then blocked with 5% BSA for 30 min at room temperature. Cells were incubated with the desired primary antibody for 4h at 1:100 dilution. After washing with PBS, samples were incubated with the desired secondary antibody at 1:100 dilution and counter stained for Phalloidin (1:100, Sigma Aldrich, Cheshire, UK) and/or PI (Vector Laboratories, Peterborough, UK) in the dark for 1h. After extensive washing, samples were mounted with mounting medium (Vector Laboratories, Peterborough, UK), and examined by confocal microscopy Eclipse TE2000-U, Nikon, Surrey, UK) at 40X magnification, numerical aperture 0.65. Primary and secondary antibodies used in the study are listed in (Table 2).

**Table 2:** Immunocytochemistry antibodies

Antigen	Dilution	Host	Supplier
**1^st^ antibodies**			
p27kip1	1:100	Rabbit	Life technologies
PKCα	1:100	Rabbit	Cell signalling
PKCι	1:100	Rabbit	Abcam
PKCζ	1:100	Rabbit	Abcam
PKCμ	1:100	Mouse	Abcam
PKCε	1:100	Rabbit	Abcam
**2^nd^ antibodies**			
Anti-rabbit 488	1:100	Goat	Life technologies
Anti-mouse 488	1:100	Goat	Life technologies

### Data and statistical analysis

Data were expressed as mean ± SEM with P<0.05 deemed statistically significant. Differences between groups were assessed using either an independent t test or one-way analysis of variance with Dunnett's or Tukey's post-hoc tests.

## SUPPLEMENTARY MATERIAL FIGURES



## Abbreviations

AMD: age-related macular degeneration
A2CE: alsterpaullone, 2-cyanoethyl
oxPOS: oxidised photo-receptor outer segment
PKC: protein kinase C
POS: photoreceptor outer segment
PVR: proliferative vitreoretinopathies
RPE: retinal pigment epithelium
ROS: reactive oxygen species
