# Supplementary material for: PKCζ-dependent upregulation of p27kip1 contributes to oxidative stress induced retinal pigment epithelial cell multinucleation
Source: Aging (Albany NY). 2017 Oct 9;9(10):2052–68. doi: 10.18632/aging.101299 (PMC5680555; doi:10.18632/aging.101299)
Supplement: Supplementary file 1 [file aging-09-2052-s001.pdf]

SUPPLEMENTAL MATERIAL

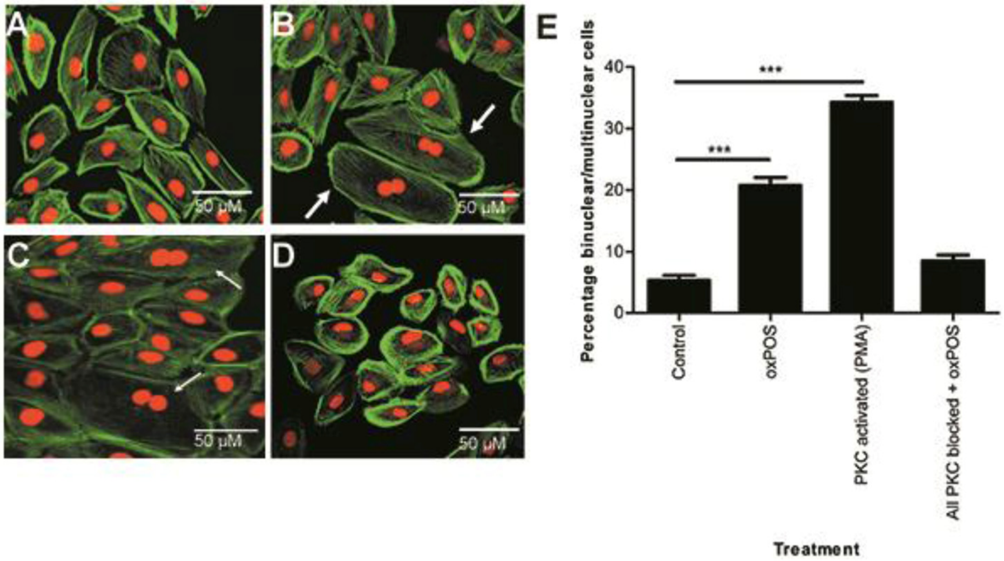

Figure S1. PKC activation induced human RPE multinucleation. (**A**) control untreated human RPE. (**B**) PMA treated human RPE. (**C**) oxPOS treated human RPE. (**D**) PKC blocked human RPE. Arrows indicate multinucleate RPE cells. (**E**) histogram showing the percentage of binucleated and multinucleated RPE cells following PKC activation and inhibition, ***, $P < 0.001$ compared to control untreated. One-way ANOVA followed by Dunnett's multiple comparison test. 50 cells were counted from three wells for each group.

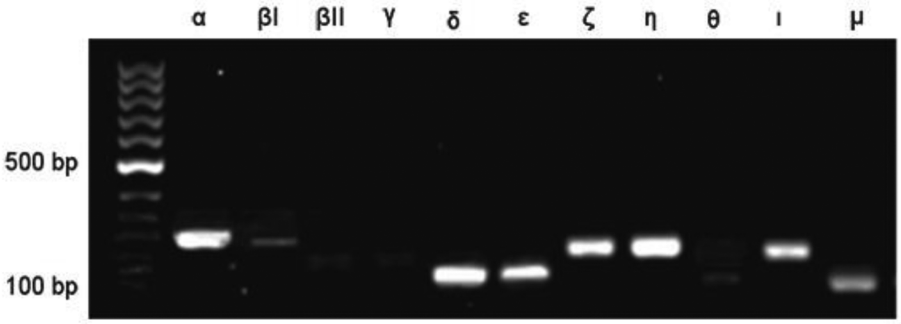

Figure S2. ARPE19 cell PCR product gel for the eleven different PKC isoforms.

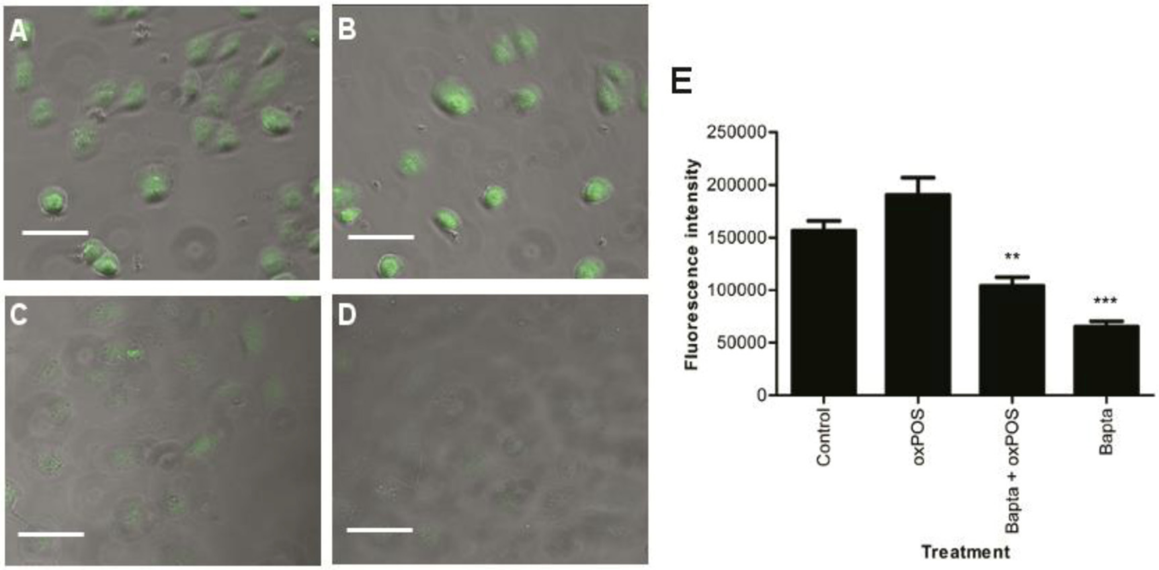

Figure S3. Confocal images confirming Ca2+ blockade using Fluo-4 live cell staining. (**A**-**B**) complete untreated control and oxPOS-treated RPE cells with strong Fluo-4 calcium staining. (**C**-**D**) BAPTA-AM +oxPOS-treated RPE and BAPTA-AM-treated control RPE cells with minimal Fluo-4 calcium iron staining. (**E**) quantification of fluorescence intensity of Fluo-4 in the BAPTA AM-treated groups; ** P <0.01, *** P <0.001 compared to the complete control. One-way ANOVA followed by Dunnett's multiple comparison test. 50 cells were counted from three wells for each group.

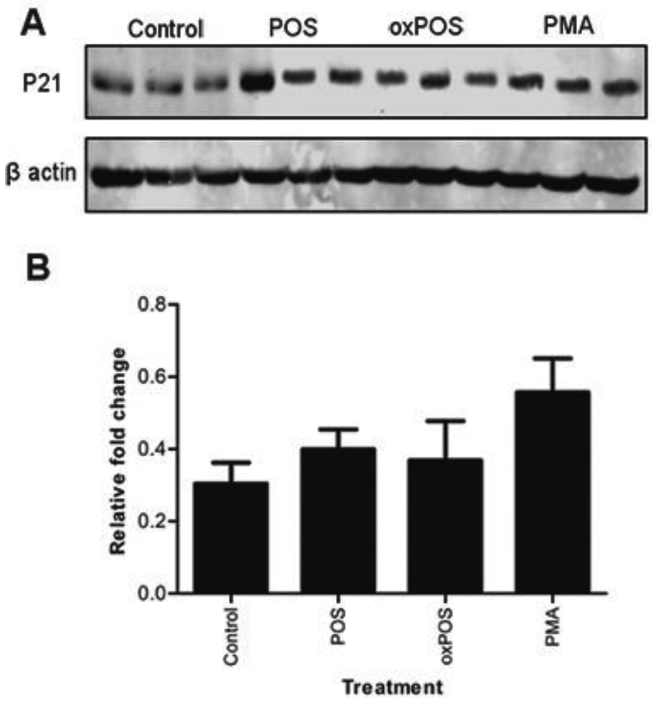

Figure S4. p21 expression in POS and oxPOS-treated RPE cells. (**A**) representative western blots from different treatment groups showing p21 at 18 KDa and β-actin at 40 KDa. Protein was separated by SDS-PAGE using a 10–15% polyacrylamide gradient. Equal amounts of protein were loaded for each treatment group and the treatment groups are indicated at the top. (**B**) quantification of p21 expression. Signals from western blots were visualised by Odyssey infrared imaging system and quantified using ImageJ analysis software (version 1.45). Signals for control were set to one and the data are represented as relative fold change *P <0.05 compared to the control untreated group. One-way ANOVA followed by Dunnett's multiple comparison test. N= 3.

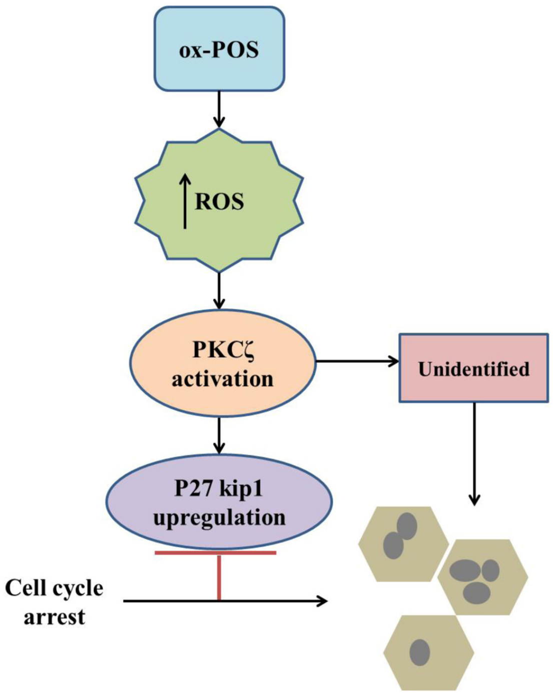

Figure S5. Proposed mechanisms of PKCζ regulation on RPE multinucleation.
